# Electricity in Dentistry

**Published:** 1888-12

**Authors:** 


ARTICLE IV.

### ELECTRICITY IN DENTISTRY.

As an instance of the rapid march of science, and its adaption to the varying needs of mankind, one would perhaps, be inclined to place electricity in the very foremost rank; not only on account of the tremendous strides which it has of late taken as an abstract science, but more particularly in view of the fact that to it we owe so many of the material comforts which we are privileged to enjoy in these modern days, when Nature's secrets are being ferreted out at a rate which would fairly have puzzled our more easy-going fore-fathers. Science has always ministered to the wants of man—to the alleviation of his sufferings, and to the amelioration of his condition. It has done much, in conjunction with art, for our own specialty; and it is, therefore, but reasonable that, in reviewing the large field of usefulness which electricity now covers, we should enquire as to any special benefits which it is capable of conferring upon dentistry, and seize upon them, in the interests both of ourselves and our patients.

One of the most formidable enemies of the dentist (especially if he be located in London) is light—or rather the absence of it—during a large portion of his working hours. To attempt delicate work in a bad light is to court failure, to experience disappointment, and to tread that borderland where loss of self-control merges into despair. The electric light is doing much to lessen the gloom of our cities, and is eminently adapted to dental requirements from its peculiar advantages of brilliancy of illumination, absence of noxious products, and the definition of daylight hues of color. To those within reach of having this beautiful lighting power "laid on," there can be no excuse for not availing themselves of the boon—except it be that of expense. We understand, however, that the current is not suitable for purposes other than lighting; but, surely this is an obstacle to be overcome in the future. To those who attempt anything beyond the casual use of a tiny oral lamp driven from a primary battery, disappointment comes sooner or later; and hosts of batteries, "guaranteed" to do the most marvelous work. have come and gone, like spectres of ill-fame. We are aware that a great amount of care and trouble in certain instances have yielded fair results, but if domestic lighting on a practical scale had been feasible from primary batteries, we should have seen them in general use long ere this. The hope of the present appears to cling to secondary batteries or accumulators, but the storage necessary for lighting on anything like an adequate scale must prove a stumbling-block to the general adoption of this system.

In the direction of motors and mallets, things are certainly more promising, for a large section of our younger operators have utilized primary batteries for these purposes for some years with a fair amount of success. It took some time to familiarize the public with the dental engine, and it is not impossible even to-day to lay one's hands on both practitioners and patients, who are strongly prejudiced against its use. In like manner, it will take some time to overcome the prejudice which exists against the employment of motors, but in future we have no doubt that they will become very general, and will be appreciated as lessening the dentist's exertions, while performing their work with greater effect, and less discomfort to the patient. Water-power has its advocates for this purpose: but, whilst fully admitting its many advantages, yet we cannot ignore the fact that the general use of electricity for motor purposes in the future must also appeal to us, as being adequate, and economical. Meanwhile, we are casting about for suitable electrical motor power, and the principle of *storage* seems to be coming to the front. Should this method prove, after reasonable trial, to fulfil our requirements, the way will have been prepared for the more general utilization of dental motors.

If opinions differ as to the way in which we should drive our engines, the same is doubly true as to the methods of introducing gold; and even those who employ mallets are sharply divided as to the particular form of blow which should be given. The advocates of the electric mallet are, we believe, on the increase; and, although it will probably never become a universal idol, yet we venture to prophesy, that it will hold its own in years to come as a reliable adjunct to cohesive gold filling. Although the form of the mallet needs much improvement, yet we are more concerned at the present moment with a suitable power for working it. All those who have had any experience of primary batteries know something of the chagrin caused by failure of power in the middle of a large filling, except reserve cells be requisitioned to help one over the stile. Our remarks on motors will also apply to mallets, and a reliable power will be hailed with much pleasure, not only as a solution of much that is perplexing, but also as a medium of making more popular an instrument which deserves the attention of every student of dentistry.—*Ex.*

